# Traumatic Chronic Subdural Hematoma: A Case Report in a Patient With Bilateral and Massive Compromise of the Frontal Lobes

**DOI:** 10.7759/cureus.77343

**Published:** 2025-01-12

**Authors:** Floricel Olimpia Villegas Amador, Luis Eduardo Nava Mata, Yazmin Cárdenas Ramos, Nemi Isabel Pérez Peña, Luis Enrique Sanchez García

**Affiliations:** 1 Transplant and Donation Department, Regional General Hospital 1 of the Mexican Social Security Institute, Querétaro, MEX; 2 Faculty of Medicine, Universidad Autónoma de Querétaro, Querétaro, MEX; 3 Surgery, Regional General Hospital 1 of the Mexican Social Security Institute, Querétaro, MEX; 4 General Practice, Regional General Hospital 1 of the Mexican Social Security Institute, Querétaro, MEX

**Keywords:** chronic subdural hematoma (csdh), cranial trauma, frontal lobe damage, intracranial arachnoid cyst, young adult male

## Abstract

Chronic subdural hematoma (CSDH) is a collection of blood in the subdural space that persists for more than three weeks. It is most commonly observed in the elderly, typically following mild to moderate cranial trauma; however, it is rare in young patients. We present the case of a 24-year-old male who presented mild cranial trauma secondary to a fall from his own height due to tonic-clonic seizures. Initially, he exhibited a transient loss of consciousness, followed by neurological improvement, with a Glasgow Coma Scale score of 15. Radiological imaging revealed extensive bilateral complicated arachnoid cysts (AC) in the frontal region, leading to craniotomy and drainage. Despite surgical intervention, follow-up imaging demonstrated rebleeding, necessitating further surgical reintervention. Surgical findings included bilateral CSDH secondary to hemorrhagic AC, as well as absence of the frontal lobes. This case represents an unprecedented source of insight into the diagnostic challenge posed by CSDH in the presence of significant developmental alterations of the frontal lobes, adding complexity to management due to potential cognitive and neurological implications.

## Introduction

Chronic subdural hematoma (CSDH) is a common neurological condition, particularly in the elderly, characterized by blood accumulation in the subdural space. The global incidence of CSDH is increasing, reflecting the aging population [1]. The pathogenesis of CSDH is multifactorial and complex, involving traumatic and inflammatory processes that promote the formation of membranes with permeable and fragile neovessels, which predispose to chronic recurrent microhemorrhages and hematoma expansion. Trauma, even minor, is the most common precipitating factor for CSDH, which usually occurs three or more weeks after the event, are bilateral in approximately 20% of cases, and are associated with higher morbidity and mortality [2]. A non-contrast cranial computed tomography (CT) scan is commonly used as the first-line imaging modality for CSDH [3]. Historically, surgical evacuation is the gold standard for managing symptomatic CSDH with burr hole craniostomy using a closed drainage system. Several different operative strategies and perioperative strategies such as the addition of burr holes, addition of cavity irrigation, position of drain, or postural position, have been described previously [4]. Despite these advances in treatment, recurrence of CSDH remains a major challenge, affecting approximately 10-20% of patients, often requiring repeat surgical intervention [1]. 

Arachnoid cysts (AC) are benign lesions characterized by the accumulation of cerebrospinal fluid (CSF)-like fluid within a duplication of the arachnoid membrane. These cysts are typically congenital and not considered neurodegenerative. AC may be detected in childhood but can also develop later in life, with potential for growth or spontaneous size reduction. While most are asymptomatic, AC can cause complications if they rupture or create a mass effect, leading to conditions like CSDH. Even small arachnoid cysts can increase the risk of CSDH after mild head trauma, particularly in young patients, where CSDH is otherwise rare [5,6].

The embryonic development of the frontal lobes is a complex process involving multiple stages of neuronal proliferation, migration and differentiation. During the first weeks of gestation, progenitor neurons are generated in the telencephalic neuroepithelium, where they begin migrating to their final positions in the developing brain [7]. Developmental disorders of the frontal lobes may include various neuronal migration disorders, such as lissencephaly, heterotopia, and polymicrogyria. These brain malformations not only impair cortical organization but can also serve as substrates for secondary pathologies. The etiology of frontal lobe developmental anomalies is multifactorial, encompassing both genetic mutations and environmental factors. Such disorders are frequently associated with other central nervous system anomalies, including corpus callosum dysgenesis and absence of the septum pellucidum, reflecting the interconnected nature of brain development [8,9]. 

Here, we present the case of a young adult with bilateral frontal lobe involvement of unknown etiology discovered during the management of a CSDH secondary to AC. This report integrates the pathological and clinical aspects of this unique presentation, focusing on the challenges in the management of CSDH in the context of concurrent developmental alterations of the frontal lobes and AC rupture.

## Case presentation

**Figure 1:**
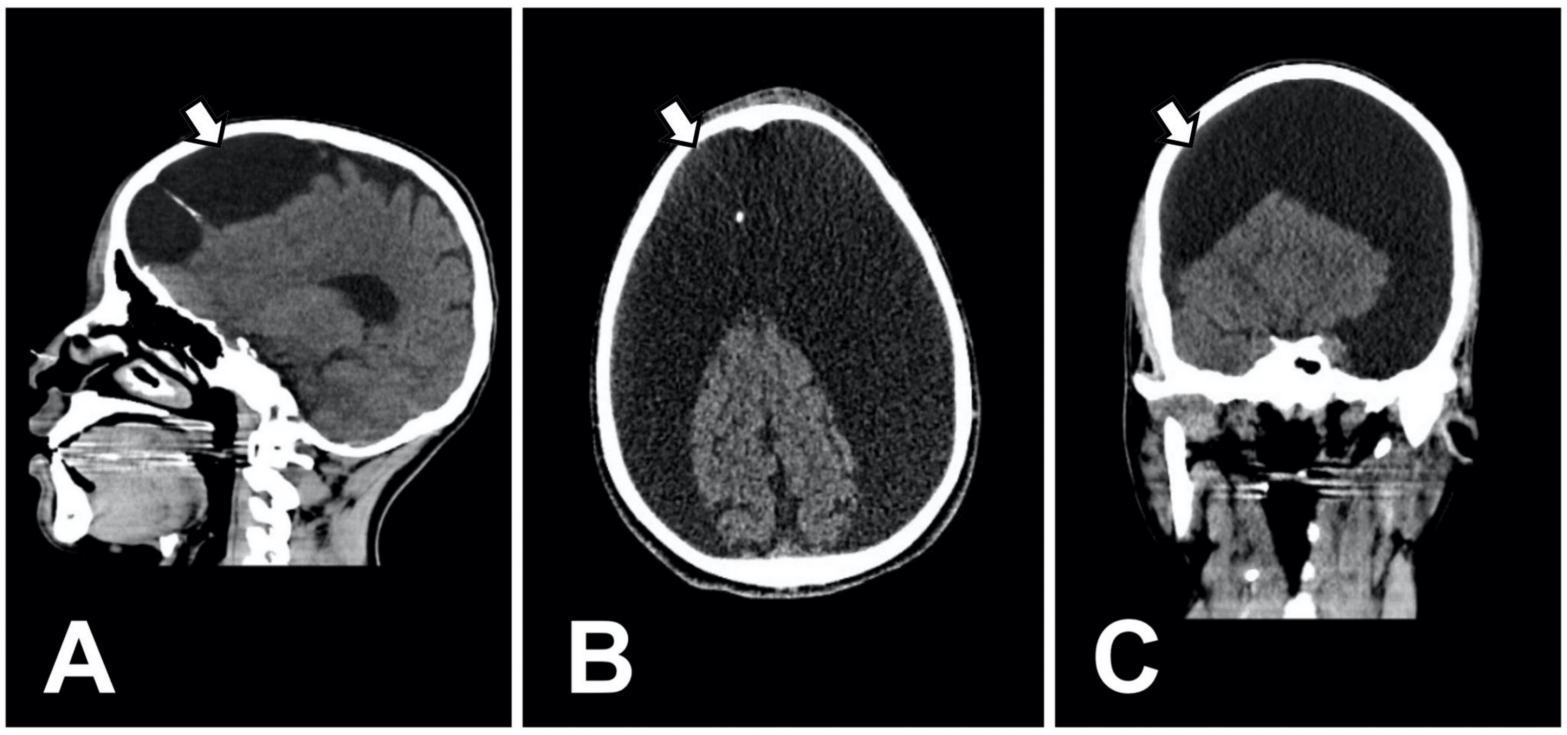
Non-contrast cranial computed tomography (CT) CT scan with sagittal (A), axial (B), coronal (C) views reveals ovoid lesions in the frontotemporal region (arachnoid cysts). No abnormalities are observed in the brainstem or cerebellum, and normal differentiation between gray and white matter is maintained.

A 24-year-old male with a history of normal neurological development during childhood presented with a two-year history of epilepsy of unknown etiology. His epilepsy was initially treated with valproic acid, but treatment was discontinued after one year due to drowsiness and lack of follow-up caused by the SARS-CoV-2 pandemic. He was admitted to the emergency department following mild cranial trauma secondary to a tonic-clonic seizure, which caused him to fall from his own height and experience a loss of consciousness. He was initiated on phenytoin therapy, and physical examination revealed neurological improvement, with a Glasgow Coma Scale score (GCS) of 15. A cranial CT scan was performed, and the patient was referred to the neurology outpatient clinic for further evaluation and management. The electroencephalogram (EEG) revealed paroxysmal frontal midline theta wave activity. CT findings revealed the presence of probable septated AC with possible left temporal lobe and frontal lobe displacement (Figure 1).

**Figure 2:**
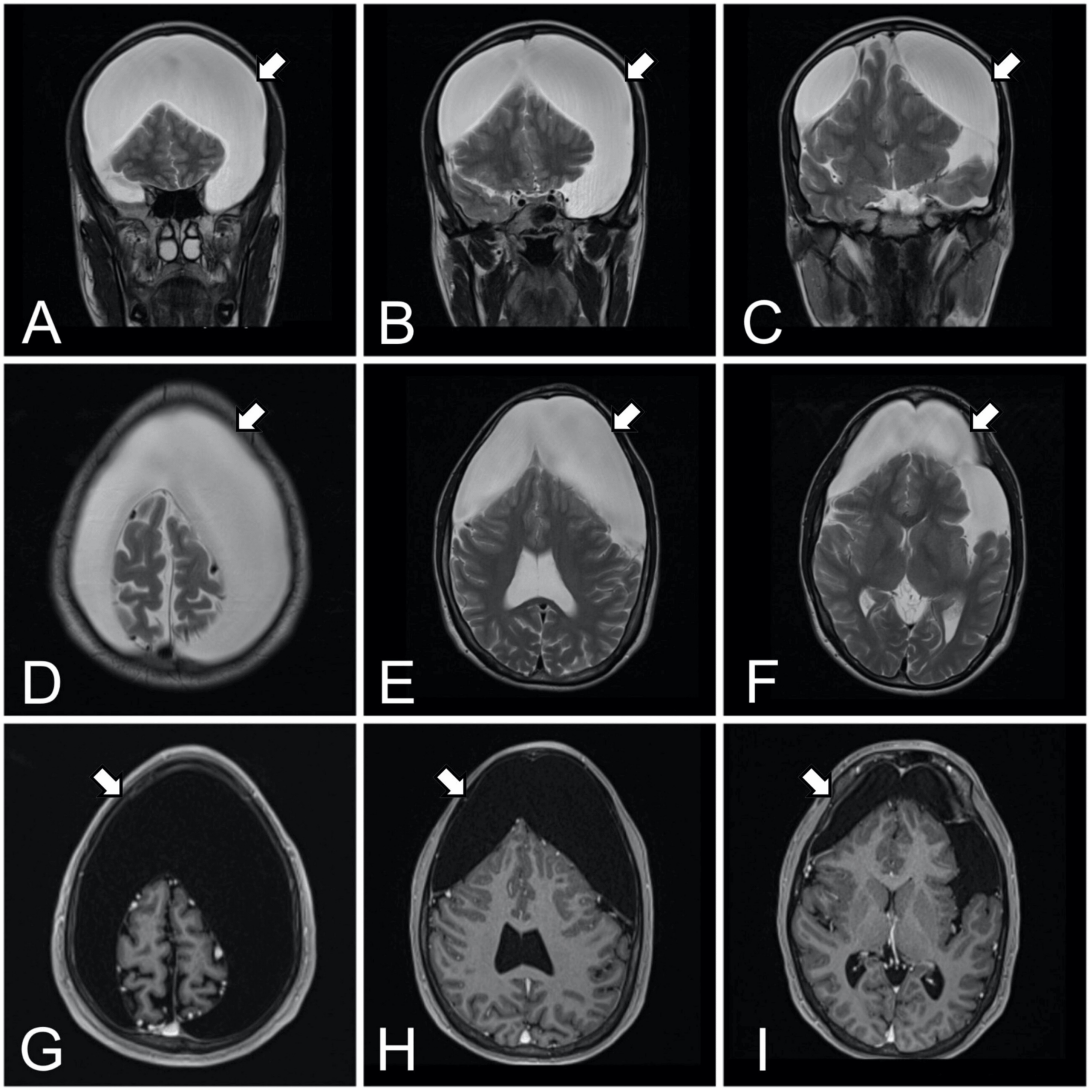
Brain Magnetic Resonance Imaging (MRI) Simple cranial MRI, T2-weighted coronal slices (A-C), T2-weighted axial slices (D-F). Contrast MRI axial slices in T1 weighting (G-I). The presence of arachnoid cysts occupying the region of the frontal lobes, hyperintense in T2, hypointense in T1, and without enhancement following contrast administration. The remaining cerebral hemispheres showed no evidence of space-occupying lesions, with normal convolution depth and well-differentiated sulci and fissures. Additionally, the cerebellum, cerebellar base, pons, and medulla were unremarkable with no abnormalities observed.

The patient was referred to the neurosurgery department. Physical examination revealed ecchymosis on the lower eyelid, reactive pupils, and a GCS of 15. No focal neurological deficits were noted, and there were no signs of increased intracranial pressure. The limbs were intact, and no seizures had been observed since the head injury. A contrast-enhanced cranial MRI was performed, which confirmed the presence of frontal arachnoid cysts. On the left side, the cyst measured 14.35 cm x 5.61 cm x 5.92 cm along the longitudinal, anteroposterior, and transverse axes, while on the right side it measured 11.33 cm x 5.11 cm x 4.78 cm (Figure 2). The patient was scheduled for bilateral parieto-temporal decompressive craniotomy with placement of bilateral subdural drains. Intraoperative findings revealed clear tension fluid upon right durotomy, absence of frontal lobe parenchyma, and a left-sided chronic subdural hematoma.

**Figure 3:**
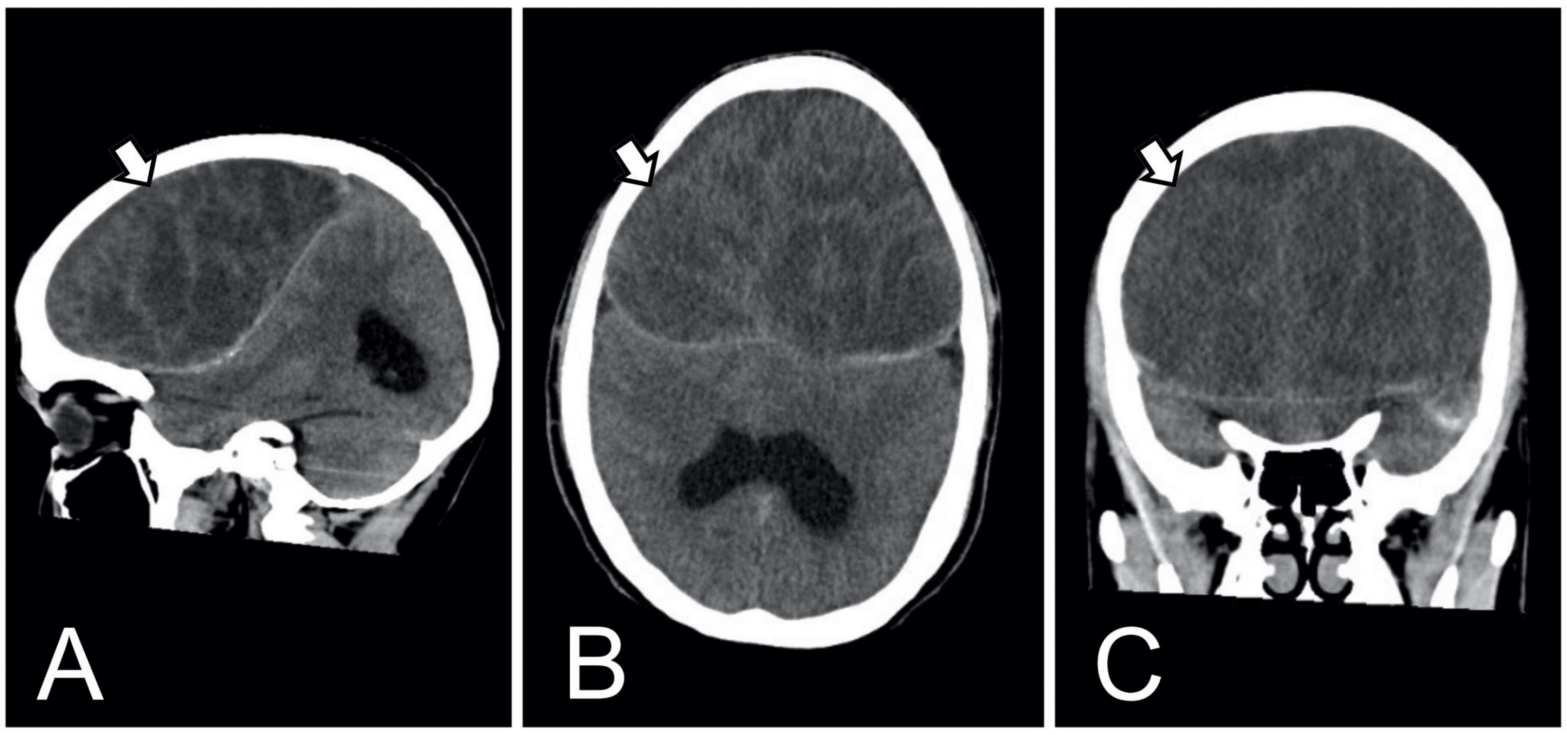
Non-contrast cranial computed tomography (CT) CT scans with sagittal (A), axial (B), and coronal (C) views. Revealed heterogeneous lesions in the frontal region. These findings were suggestive of chronic membranes with evidence of rebleeding and a mass effect.

**Figure 4:**
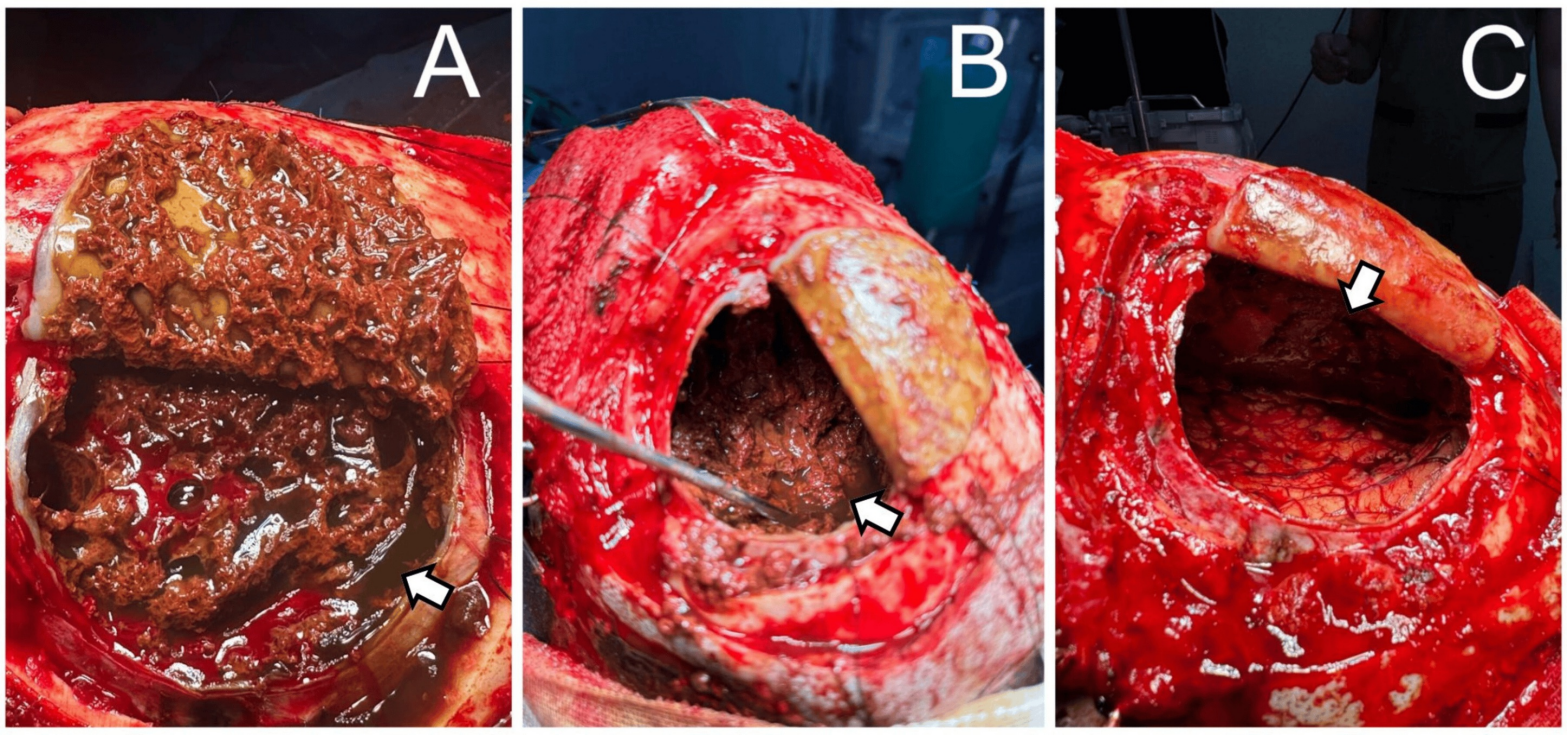
Surgical findings The surgical image demonstrates the opening of the dura mater, revealing thick vascular membranes and a consolidated hematoma with a liquid component resembling "burnt motor oil" within the subdural space (A). Aspiration, hemostasis, and removal of a dense, fibrous vascular membrane adherent to the brain parenchyma, consistent with a hemorrhagic arachnoid cyst, were successfully performed (B). There was an absence of brain parenchyma corresponding to the frontal lobes (C).

**Figure 5:**
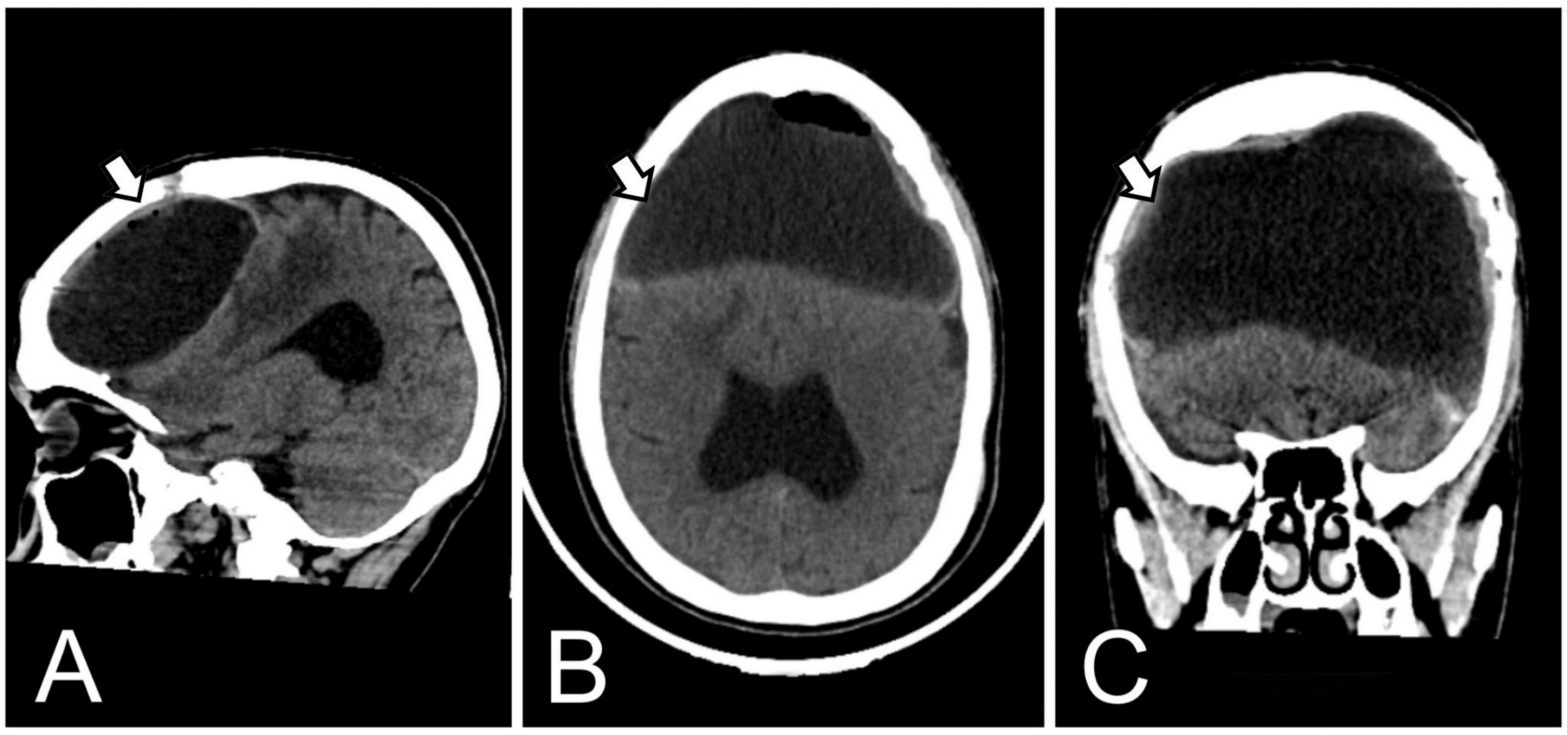
Postoperative Non-contrast Cranial Computed Tomography (CT) CT scans with sagittal (A), axial (B), and coronal (C) views. The images revealed the presence of arachnoid cysts occupying the frontal region, without evidence of rebleeding.

In the immediate postoperative period, the patient demonstrated satisfactory neurological progression, with no alterations in executive or cognitive functions. The drainage was removed as it was clear and within controlled limits. The patient was discharged home due to clinical improvement. Seizures remained well-controlled with phenytoin and levetiracetam for the next two years. During this period, the patient was diagnosed with generalized anxiety disorder and major depressive disorder. Subsequently, a follow-up CT was performed, revealing heterogeneous lesions consistent with rebleeding and a mass effect (Figure 3). As a result, the decision was made to admit the patient for a second craniotomy. The surgical findings revealed a bilateral chronic subdural hematoma with exacerbation, associated with mixed vascularized membranes. A thick, vascularized fibrous membrane was found adherent to the brain inside the arachnoid cyst. Additionally, there was a loss of brain volume in the region corresponding to the frontal lobes (Figure 4). The patient was transferred to the intensive care unit for the management of complications. It was later decided to discharge the patient in the late postoperative period, as neurological progress was satisfactory and a follow-up CT showed no evidence of rebleeding (Figure 5). The patient remained asymptomatic with adequate seizure control, and a follow-up appointment was scheduled for five months to monitor progress and ensure continued management of the condition.

## Discussion

AC in young adults may predispose individuals to develop CSDH with or without a history of cranial trauma. Benek et al. identified complicated AC in 10 out of 285 CSDH patients, with eight located in the middle cranial fossa [10]. In contrast, our case involved an atypical location in the anterior cranial fossa, occupying the entire territory corresponding to the frontal lobes. Most AC are asymptomatic, but when they grow or become complicated they may cause headache, seizures or other neurological symptoms [5,10]. In this particular case, the patient presented with epilepsy characterized by tonic-clonic seizures. Although antiepileptic drug prophylaxis is commonly used, the evidence for its effectiveness in preventing seizures in patients with CSDH is controversial and not well established [11].

Surgical management of CSDH in young adults typically involves craniotomy or craniectomy with trepanation, yielding favorable outcomes with low recurrence rates [12,2]. Pacheco-Barrios et al. found that patients who underwent trepanation with drainage had a lower seizure risk compared to those treated with craniotomy [13]. In our case, craniotomy was performed, resulting in a favorable outcome without postoperative seizures. Benek et al. suggested surgery for CSDH patients with a diameter >10 mm, while those with smaller hematomas were managed conservatively. Follow-up neuroimaging in their study showed resolution of both the hematoma and AC size over time, with no neurological complications [10]. Recent innovations in CSDH treatment, such as middle meningeal artery embolization, show promise in reducing recurrence and improving neurological outcomes, either as a single treatment or in combination with surgical evacuation [14].

Cognitive deficits are common in CSDH patients but tend to improve post-surgery. A systematic review reported that 61% of patients had cognitive impairment before treatment, decreasing to 18% postoperatively [15]. However, a recent study found that CSDH patients still exhibited significantly lower cognitive performance compared to healthy controls even three months after surgery, underscoring the need for continuous cognitive follow-up [16].

The frontal lobe is crucial for executive functions, impulse control, social behavior, and decision-making, playing a key role in cognitive abilities. Damage to this region can lead to significant deficits in these functions, while other cognitive abilities may remain relatively intact [17]. To date, only one other study, by Ibáñez et al., has documented massive developmental involvement of the frontal lobes. This study reported almost complete loss of frontal connections in a pediatric case [17]. While agenesis has been observed in other brain regions, such as the cerebellum, temporal lobes, and corpus callosum, "frontal lobe agenesis" is not a widely recognized or studied term in current medical literature. Our case represents an additional contribution to this rare condition. However, it is constrained by the absence of tools like Diffusion Tensor Imaging (DTI) and Functional Magnetic Resonance Imaging (fMRI), which could assess both structural and functional brain connectivity. These technologies would provide a clearer understanding of the compromised and preserved functions, as demonstrated in the Ibáñez et al. study. In that case, a pediatric patient with bilateral frontal lobe involvement exhibited significant impairments in functions such as abstraction, attention and cognitive control, while sensorimotor and higher-level cognitive functions, such as consciousness, language, memory, and social interaction, remained relatively intact due to compensatory contributions from posterior brain regions [17].

In our case, neurological development during childhood and adolescence was normal until the onset of seizures, likely related to the complicated AC. Despite surgical intervention for the CSDH, no significant clinical changes related to frontal lobe function were observed. This highlights the importance of comprehensive neurological evaluations in patients with significant frontal lobe involvement. Especially in the presence of CSDFH secondary to complicated AC, as these conditions not only alter brain structure, but may also affect cognitive and executive functions. Thus, while resolving CSDH is crucial for alleviating the subdural condition, it may not always lead to clinical improvement in cognitive functions in patients with massive frontal lobe involvement. This represents a challenge in both the diagnosis and treatment of CSDH, complicating the prediction of postoperative neurological outcomes.

## Conclusions

This case describes a CSDH secondary to a hemorrhagic AC in a patient with massive bilateral frontal lobe involvement, who surprisingly exhibits remarkable preservation of all functions despite significant underlying anatomical damage. AC are congenital intracranial lesions that can complicate into CSDH, particularly after minor cranial trauma. The rare combination of massive frontal involvement with CSDH presents significant challenges in clinical evaluation and management, complicating the prediction of postoperative outcomes. The literature highlights the importance of a multidisciplinary approach that includes not only surgical intervention for CSDH resolution but also ongoing neurocognitive monitoring to optimize clinical outcomes. Further research is essential to improve the management of these complex cases and develop more effective therapeutic strategies in these types of challenging conditions.
